# Post-COVID-19 syndrome and quality of life impairment in severe COVID-19 Mexican patients

**DOI:** 10.3389/fpubh.2023.1155951

**Published:** 2023-05-15

**Authors:** Carla Marina Román-Montes, Yesenia Flores-Soto, Guillermo Arturo Guaracha-Basañez, Karla María Tamez-Torres, José Sifuentes-Osornio, Ma. Fernanda González-Lara, Alfredo Ponce de León

**Affiliations:** ^1^Clinical Microbiology Laboratory, Instituto Nacional de Ciencias Médicas y Nutrición Salvador Zubirán, Mexico City, Mexico; ^2^Infectious Diseases Department, Instituto Nacional de Ciencias Médicas y Nutrición Salvador Zubirán, Mexico City, Mexico; ^3^Emergency Department, Instituto Nacional de Ciencias Médicas y Nutrición Salvador Zubirán, Mexico City, Mexico; ^4^General Direction, Instituto Nacional de Ciencias Médicas y Nutrición Salvador Zubirán, Mexico City, Mexico

**Keywords:** post-COVID-19 syndrome, long-COVID-19, quality of life, chronic COVID-19 syndrome, severe COVID-19, Mexican

## Abstract

**Introduction:**

Post-COVID-19 syndrome (PCS) usually occurs 3 months after the onset of COVID-19 with a symptom duration of at least 2 months without an alternative diagnosis.

**Objective:**

This study aimed to describe the prevalence, characteristics, and impact on the quality of life (QoL) of post-COVID-19 syndrome in patients with a history of hospitalization for COVID-19.

**Materials and methods:**

We conducted a cross-sectional study. Patients who required hospitalization due to COVID-19 between March 2020 and October 2021 were invited to answer a PCS questionnaire and the EQ-5D instrument. A total of 246 patients were included: 187 (76%) met the definition of PCS and 54% were men, with a median age of 50 years (IQR 41–63).

**Results:**

From 187 patients with PCS, the median time to symptom onset after hospital discharge was 1 day (IQR 1–20), and the median symptom duration was 150 days (IQR 90–225). A total of 27 different symptoms were reported; the most frequent were difficulty concentrating (81%), dyspnea (75%), arthralgia (71%), fatigue (68%), and hair loss (60%). Some symptoms, such as difficulty concentrating, arthralgia/myalgia, and hair loss, were more prevalent in women with PCS. Patients with PCS had a higher frequency of tobacco smoking (37 vs. 4%, *p* = 0.02) and increased severity of lung involvement in the initial chest tomography (75 vs. 58%, *p* = 0.01) than those without PCS. Patients with PCS were less likely to receive antivirals (15.5 vs. 27%, *p* = 0.04). No difference between ICU admission, mechanical ventilation, and length of hospital stay was found. Patients with PCS had a lower visual analog scale result for EQ-5D vs. those without (80 [IQR 70–90] vs. 89.5 [IQR 75–90], *p* = 0.05). All five QoL dimensions were affected in PCS patients, showing increased pain/discomfort (67 vs. 39%, *p* = < 0.001), difficulties in performing usual activities (39.2 vs. 20.3%, *p* = 0.03), and anxiety/depression (57.5 vs. 37%, *p* = 0.02).

**Conclusion:**

PCS occurred in 76% of hospitalized patients with prolonged duration and QoL impairment. Neurological symptoms such as difficulty concentrating were the most frequent symptoms. Timely diagnostic and therapeutic interventions are required.

## Introduction

Coronavirus disease-19 (COVID-19) is still a critical comorbidity and mortality cause worldwide. SARS-CoV-2 infection no longer carries the same risks of adverse outcomes as it did in the early months of the pandemic because of the vaccines and the new subvariants of SARS-CoV-2 with a diverse rate of transmission and virulence (1). Post-COVID-19 syndrome (PCS) was defined by the World Health Organization (WHO) in late 2021 as symptoms occurring in individuals within 3 months of a history of probable or confirmed SARS-CoV-2 infection, with at least 2 months that cannot be explained by an alternative diagnosis (2). Common symptoms of PCS include shortness of breath, fatigue, difficulty thinking or concentrating (referred to as “brain fog”), changes in smell and taste, sleep problems, and hair loss. Before this definition, different terms such as long COVID syndrome, persistent post-COVID syndrome, and post-acute COVID-19 syndrome were used. Symptoms may be new onset following initial recovery or persistent since the initial COVID-19 episode. Symptoms may also fluctuate or relapse over time.

A meta-analysis reported a high prevalence of up to 80% (95% CI 65–92%) (3). Other cohorts report a lower prevalence, such as 32.6% in Michigan, USA, with limitations such as the study date and lack of PCS definition (4). Another study in Wuhan, China, using questionnaires, physical examination, 6-min walk tests (6MWT), laboratory tests, pulmonary function tests (PFTs), and high-resolution computed tomography described that 76% had at least one symptom. Lopez-León et al. described more than 50 symptoms as part of PCS; among them, the most frequent were fatigue (58%), headache (44%), attention disorder (27%), hair loss (25%), and dyspnea (24%) (3, 5). Other reviews and meta-analyses in the UK also found fatigue (37%) as the most prevalent, followed by dyspnea (6).

The quality of life (QoL) definition encompasses individual perceptions of their position in life in the context of the culture and value systems concerning goals, expectations, standards, and worries (7). Hence, QoL is used as a general predictor of health and is essential to understand the repercussions of COVID-19 on physical health status, social restrictions, and psychological states. Several tools measure the QoL; some are generic, such as SF-36 (36-item Short-Form Health Survey) and EQ-5D (EuroQol-5 Dimension), and they are used to assess multiple domains of the health and wellbeing of the patient. Specific tools are also used in diseases such as rheumatoid arthritis and metabolic disorders (8).

In other pandemic scenarios, such as influenza, QoL impairment was described (9). After the COVID-19 pandemic, it has been published that survivors' QoL is generally affected, particularly in patients with PCS (10–12).

In this study, we aimed to describe the prevalence of PCS, the frequency of symptoms, and the impact on the QoL of patients with an initial episode of severe or critical COVID-19.

## Methods

### Patients and data collection

**Figure 1:**
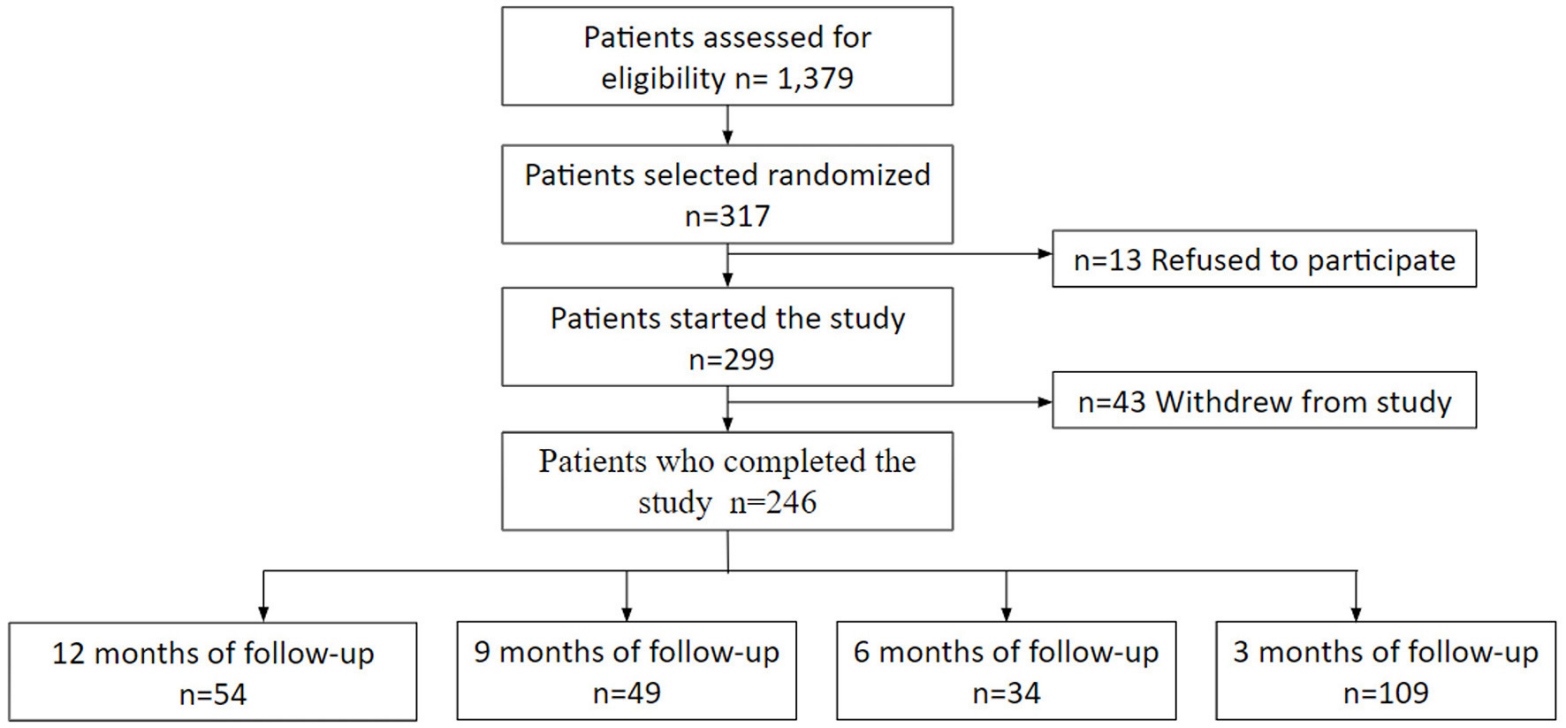
Pacients flowchart. Comments: Patients assessed for eligibility were patients who were hospitalized in the chosen period and who were discharged home.

A retrospective, cross-sectional study was done. We found 1,379 patients ≥18 years of age hospitalized with COVID-19 between 1st July 2020 and 31st December 2021. Among these, 317 who had been hospitalized 3, 6, 9, and 12 months before were randomly selected and invited to answer an adapted questionnaire to identify the presence of PCS and EQ-5D. Among 312 patients who were invited to participate, only 246 patients were included who then answered both the questionnaires (Figure 1).

### Research tools and instruments

The content of the PCS questionnaire was adapted from the questionnaire used by Huang et al. on a group of Chinese patients in 2020. A committee comprising a rheumatologist and an infectious diseases specialist performed a translation and adaptation to suit Mexican patients from the original version. The latter is a questionnaire that has been validated in multiple populations worldwide and showed good psychometric properties in Mexico (13); the first part is a visual analog scale, and the second part corresponds to five domains: mobility, self-care, usual activities, pain/discomfort, and anxiety/depression, with three possible response options: no problems, some problems, or extreme problems. The visual analog scale was reported as 0–100, where 0 represents the worst imaginable health and 100 the best imaginable health; the five domains were dichotomized into not affected (answer “no problems”) and affected (answers “some problems,” or “extreme problems”) (14). All reported symptoms, duration, and impact on QoL were recorded.

### Definitions

The WHO definition of PCS was used to classify patients into two groups: PCS and no-PCS for comparison (2). Regarding COVID-19 acute episode characteristics, we described severity using the NIH classification. We also describe the clinical, laboratory, and computed tomography (CT) characteristics and the treatment received. Definitions of the compatible or indeterminate chest CT were according to the Radiology Society of North America Expert Consensus (15). In the vaccination record, we considered only those who had a first full scheme before the COVID-19 episode.

### Statistics

Sample size calculation estimated 246 subjects considering a prevalence of 80% (2). All analyses were performed in STATA v 14.0 (StataCorp., College Station, TX, USA). Baseline characteristics are reported with descriptive non-parametric statistics, bivariate comparisons were made with the **X**^2^ test, Student's *T*-test, or Mann–Whitney's *U*-test, as appropriate, and a two-tailed *p-*value of < 0.05 was considered significant.

### Ethics

The protocol was approved by the local research and ethics committee (Local Ref Nr. 3692). Participants' data privacy was preserved during the study. Digital informed consent was signed and kept for record.

## Results

The prevalence of PCS in hospitalized patients with severe or critical COVID-19 was 76% (*n* = 187). Table 1 shows the demographic and clinical characteristics of the initial COVID-19 episode among groups. Patients with PCS had a median age of 55 years (IQR 41–63), and 54% (*n* = 101) were men. We found no statistical differences in obesity and overweight in both groups, and BMI at the time of acute COVID-19 was a median of 27.74 kg/m^2^ (IQR 25.31–32.39) vs. 29.41 kg/m^2^ (IQR 26.12–34.6). Smoking was more frequently reported in the PCS group (19.7 vs. 6.7%, RR 1.23; 95% CI 1.08–1.40, *p* = 0.02). Other comorbidities were not statistically different between the groups.

**Table 1:** Demographic and clinical characteristics of patients with and without post-COVID-19 syndrome.

**Characteristics**	**General *n =* 246 (100%)**	**PCS *n =* 187 (76%)**	**Without PCS *n =* 59 (24%)**	***p* bivariate**
Male sex	135 (54.87)	101 (54)	34 (58)	0.62
Age, median (IQR)	52.5 (41–64)	55 (41–63)	50 (39–69)	0.55
Obesity	106 (43)	86 (46)	20 (34)	0.10
Overweight	93(38)	66 (35)	27 (45)	0.14
Hypertension	82 (33)	61 (33)	21 (35.5)	0.67
Type 2 diabetes	56 (23)	42 (22)	14 (24)	0.83
Chronic kidney disease	15 (6)	9 (4.8)	6 (10.1)	0.13
Rheumatic disease	16 (6.5)	13 (7)	3 (5)	0.61
Solid cancer	6 (2.4)	4 (2)	2 (3)	0.06
Hematologic cancer	2 (0.8)	1 (0.5)	1 (2)	0.42
COPD	4 (2)	4 (2)	0	0.57
Asthma	7 (3)	6 (3)	1 (2)	0.54
Immunosuppression	23 (9)	15 (8)	8 (13.5)	0.20
HIV infection	4 (2)	2 (1)	2 (3)	0.24
Ischemic cardiopathy	10 (4)	8 (4.2)	2 (3.3)	0.76
Smoking	41 (17)	37 (20)	4 (7)	**0.02**
COVID-19 vaccine	23 (9)	18 (10)	5 (8)	0.79

PCS, Post-COVID-19 syndrome; IQR, interquartile range; COPD, chronic obstructive pulmonary disease; HIV, human immunodeficiency virus.

Table 2 shows the characteristics of the index hospitalization. More than 90% in both groups had a chest CT scan compatible with COVID-19 (PCS 97% (181/187) vs. without PCS 92% (54/59), *p* = 0.08). The remaining percentage in both groups was described as indeterminate for COVID-19.

**Table 2:** Index hospitalization for COVID-19 among patients with and without PCS.

**Characteristics**	**General *n =* 246 (100%)**	**PCS *n =* 187 (76%)**	**Without PCS *n =* 59 (24%)**	***p* bivariate**
COVID-19 compatible chest CT	235 (96)	181 (97)	54 (92)	0.08
Severe lung involvement in chest CT	174 (71)	140 (75)	34 (58)	**0.01**
SatO2 (%), median (IQR)	83 (75–87)	82 (74–86)	85 (82–88)	**0.002**
PaO2/FiO2 ratio	164.75 (92.13–251)	155.32 (92.44–251)	177.81 (89.11–252.38)	0.90
Intensive care unit admission	76 (31)	62 (33)	14 (24)	0.17
Invasive mechanical ventilation	73 (30)	59 (32)	14 (24)	0.25
Steroids for COVID-19	239 (97)	183 (98)	56 (95)	0.23
Antiviral for COVID-19	45(18)	29(15.5)	16(27)	**0.04**
Empirical antibiotic	48 (20)	36 (19)	12 (20)	0.81
Hospital length stay (days) median (IQR)	10 (6–20)	10 (6–21)	9 (5–19)	0.20
C Reactive protein, (mg/dL), median (IQR)	11.13 (6.01–18.8)	11.91 (6.66–19.26)	9.45 (5.07–17.21)	0.26
Leucocytes (x 10^3^/μL), median (IQR)	8350 (5900–12300)	8600 (6100–12600)	7800 (5300–10800)	0.12
Lymphopenia (<1.0 × 10^3^/μL)	176 (71.5)	131 (70)	45 (76)	0.35
D dimer (ng/mL), median (IQR)	699 (446–1191)	704.5 (438–1168)	682 (523–1245)	0.33
Ferritin (ng/mL), median (IQR)	566.05 (260.95–1060.1)	592.75 (280.8–1088)	519.8 (192–879)	0.15
CPK (U/L), median (IQR)	84 (43.5–161)	79 (37–159)	101 (54–190)	0.11
Lactic dehydrogenase (U/L), median (IQR)	329 (262–437)	327 (262–438)	333.5 (265–420.5)	0.97
Fibrinogen (mg/dL), median (IQR)	614 (462–767)	635 (479–776)	490.5 (429–710)	**0.006**

PCS, post-COVID-19 syndrome; CT, computed tomography; IQR, interquartile range; SatO^2^, oxygen saturation; PaO^2^, partial pressure of arterial oxygen; FiO^2^, fraction of inspired oxygen; CPK, creatine phosphokinase. Bold values are values <0.05, which means that they are statistically significant.

During the initial episode of COVID-19, PCS patients had low room air SatO2 levels (oxygen saturation) (82% IQR 74–86 vs. 85% IQR 82–88, *p* = 0.002), and their chest CT scans had severe lung involvement more frequently (75 vs. 58%, *p* = 0.01). Furthermore, 97% (182/187) of PCS patients had severe COVID-19 as per NIH classification; 33% (*n* = 62) of patients in the PCS group were admitted to the intensive care unit (ICU), and 32% (*n* = 59) required mechanical ventilation at any time during their hospitalization, with no statistical differences between groups.

**Figure 2:**
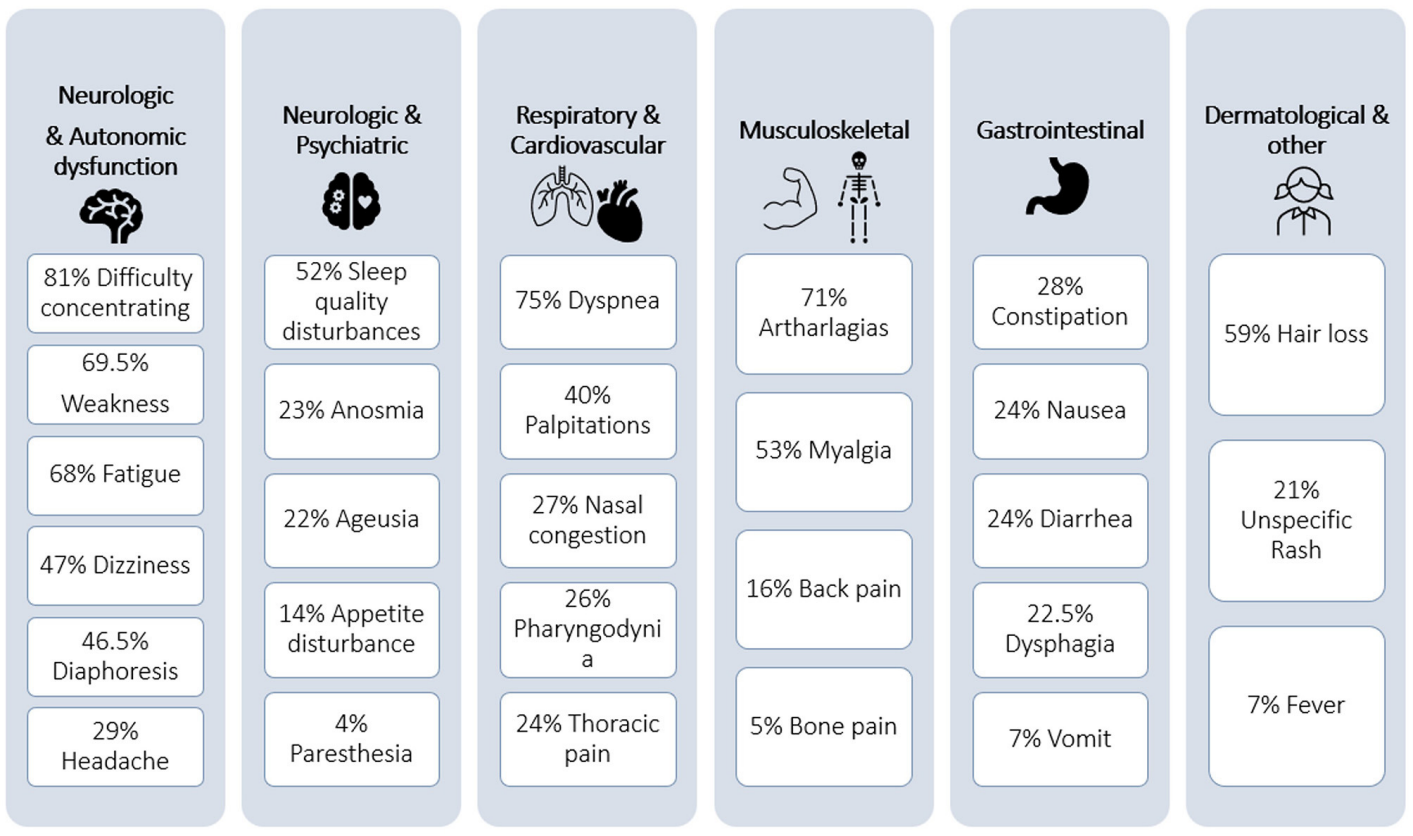
Symptoms in post-COVID-19 syndrome classify into clusters.

The most frequent symptoms of PCS were difficulty concentrating in 81% (*n* = 152), dyspnea in 75% (*n* = 141), arthralgias in 71% (*n* = 132), weakness in 69.5% (*n* = 130), fatigue in 68% (*n* = 127), hair loss in 60% (*n* = 112), myalgia in 53% (*n* = 99), sleep disturbances in 52% (*n* = 97), dizziness in 47% (*n* = 88), and palpitations in 41% (*n* = 76). A total of 27 different symptoms were described. We decided to classify them into clusters by system (Figure 2). The median time between hospital discharge and symptom onset was 1 day (IQR 1–20 days), and the median symptom duration was 150 days (IQR 90–225 days). When comparing smoking with respiratory symptoms such as dyspnea, we do not find differences, and only 18% of patients with dyspnea are smokers (*p* = 0.42). Among female patients with PCS, difficulty concentrating (87 vs. 76%, *p* = 0.05), arthralgias (79 vs.63%, *p* = 0.02), hair loss (82.5 vs. 40%, *p* ≤ 0.0001), myalgia (63 vs. 44.5%, *p* = 0.01), and dizziness (56 vs. 39%, *p* = 0.02) were more frequent compared to their male counterparts (Table 3).

**Table 3:** Frequency and differences of PCS symptoms between men and women.

**Symptoms**	**General PCS *N =* 187 (100%)**	**Males with PCS *N =* 101 (54%)**	**Females with PCS *N =* 86 (46%)**	***p* bivariate**
Difficulty concentrating	152 (81)	77 (76)	75 (87)	**0.05**
Dyspnea	140 (75)	70 (69)	70 (81)	0.06
Arthralgias	132 (71)	64 (63)	68 (79)	**0.02**
Weakness	130 (69.5)	67 (66)	63 (73)	0.30
Fatigue	126 (67)	64 (63)	62 (72)	0.20
Hair loss	111 (59)	40 (40)	71 (82.5)	**<0.0001**
Myalgia	99 (53)	45 (44.5)	54 (63)	**0.01**
Sleep disturbances	98 (52)	50 (49.5)	48 (56)	0.39
Dizziness	87 (46.5)	39 (39)	48 (56)	**0.02**
Palpitations	75 (40)	35 (35)	40 (46.5)	0.09

Bold values are values <0.05, which means that they are statistically significant.

**Figure 3:**
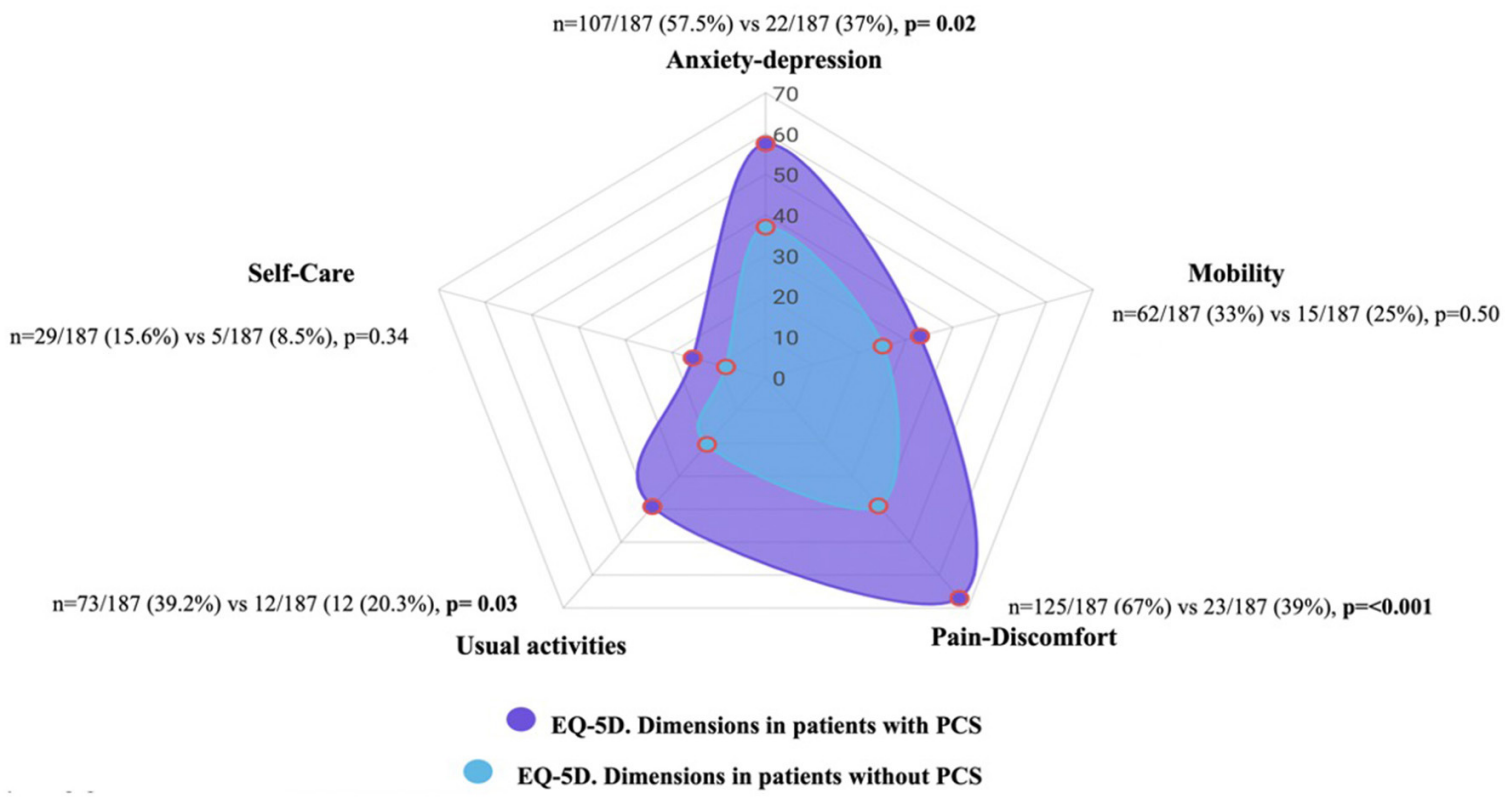
Distribution of the dimensions affected in EQ-5D.

Regarding health status and QoL, 63% (*n* = 117) of PCS patients described their health status as “worse” than before COVID-19 (OR 9.2, 95% CI 4.1–22.6, *p* ≤ 0.0001). In the EQ-5D instrument, we found disturbances in all five domains; the pain and discomfort domain was the most affected in PCS at 65.5 vs. 39% without PCS (*p* < 0.001). Also, patients with PCS were referred to a worse QoL (visual analog scale) compared to those without PCS [80 IQR (70–90) vs. 89.5 (75–90), *p* = 0.05]. Affected domains are described in Table 4 and Figure 3.

**Table 4:** Distribution of the VAS and dimensions of the EQ-5D among patients with and without PCS.

**Dimensions in EQ5D**	**General *n =* 246 (100%)**	**PCS *n =* 187 (76%)**	**Without PCS *n =* 59 (24%)**	***p* bivariate**
Visual analog scale, median (IQR)	80(70–90)	80 (70–89)	89.5 (75–90)	**0.05**
**Mobility**				0.5
-No problems	168 (69)	124 (67)	44 (75)	
-Some problems	73 (30)	59 (32)	14 (24)	
-Confined to bed	4 (2)	3 (2)	1 (2)	
**Self-care**				0.3
-No problems	211 (86)	157 (84)	54 (91.5)	
-Some problems	32 (13)	27 (14.5)	5 (8.5)	
-Unable to	2 (0.8)	2 (1.0)	0	
**Usual activities**				**0.03**
-No problems	161 (66)	114 (61)	47 (80)	
-Some problems	82 (33.5)	70 (38)	12 (20)	
-Unable to	2 (0.8)	2 (1)	0	
**Pain/discomfort**				**<0.001**
-None	99 (40)	63 (34)	36 (61)	
-Moderate	133 (54)	115 (61.5)	18 (30.5)	
-Extreme	14 (6)	9 (5)	5 (8.5)	
**Anxiety/depression**				**0.02**
-None	115 (47)	78 (42)	37 (63)	
-Moderate	120 (49)	100 (54)	20 (34)	
-Extreme	10 (4)	8 (4)	2 (3)	

PCS, post-COVID-19 syndrome; EQ-5D, EuroQol-5 Dimensions; IQR, interquartile range. Bold values are values <0.05, which means that they are statistically significant.

## Discussion

We found a 76% prevalence of post-COVID-19 syndrome in patients hospitalized for severe or critical SARS-CoV-2 infection. This rate is comparable to a systematic review describing over 50 symptoms but lower. Of note, the WHO definition we used had not been published at that time (2, 3). The definition used in the systematic review considered symptoms, signs, or abnormal clinical parameters persisting 2 or more weeks after COVID-19 onset that do not return to a healthy baseline; but given our prevalence, the defining factor does not have much impact. According to the literature, the prevalence of PCS ranges between 5 and 50%. Some extensive surveys describe a prevalence of 39%, including infections by different SARS-CoV-2 variants (16). Therefore, the variability is due to various factors: the definition of PCS, the hospitalized and non-hospitalized population, the variants, and even other additional factors such as vaccination or the treatments received.

In addition, we found 27 different symptoms as well as slightly more than half of those described in other research may be due to the strategies of searching for or questioning the presence of symptoms; the questionnaire and the interview strategy can influence the finding of more or fewer symptoms.

A few publications on PCS prevalence in Mexico were found. In a study from Guanajuato, Mexico, Muñoz-Corona et al. reported 75.9% of persistent symptoms in COVID-19 patients at 90 days of hospital discharge (17). In a study from Zacatecas, Mexico, long-term symptoms were found in 85% of patients; however, it was carried out in 2020 with a different PCS definition (18).

Some studies have associated older age and women with a higher risk for PCS (19, 20), but we did not find significant differences in age between groups nor in the proportion between sexes; this last finding may be associated with our hospitalized cohort comprising more men, with the male sex being at higher risk of severe COVID-19. However, we found some differences in the frequency of symptoms according to sex; difficulty concentrating, arthralgia, myalgia, dizziness, and hair loss are more frequent and with statistical significance in women. Fernandez DPC et al. also described that some PCS symptoms are more frequent in women such as fatigue, dyspnea, hair loss, ocular problems, depression, and poor sleep quality (20).

Fatigue is one of the predominant symptoms in PCS (21); however, respiratory manifestations, such as dyspnea, persistent cough, and chest pain, remain frequent and presumably associated with the lungs as the primary site of infection (3). Our patients referred to difficulties with concentration and attention, the so-called “brain fog,” as the main neurological complaint, with a higher than reported frequency. This has been linked to direct viral damage to the limbic system after entering through the nasal sensory cells (22). Other theories explaining cognitive abnormalities include direct neuronal infection and autoimmune/inflammatory CSF and brain tissue abnormalities (23). Ongoing studies, at our institution, found a prevalence of psychopathological PCS manifestations, memory complaint, and mild cognitive impairment a year after the acute COVID-19 episode, in 42, 45, and 30%, respectively (unpublished data, personal communication from Flores-Silva F.) The frequency of neurological symptoms during acute COVID-19 might explain the high prevalence of these symptoms. Another study from our center found that up to 65% of patients hospitalized for COVID-19 had neurological symptoms on admission, and 15% developed some neurological event, such as seizures, delirium, altered alertness, or weakness during hospitalization (24). Moreover, Wong-Chew et al. showed that the most frequent post-COVID-19 symptoms were neurological (25).

Interestingly, some overlapping with the ME/CFS (myalgic encephalomyelitis/chronic fatigue syndrome) pathogenesis has been found. Other hypotheses explaining PCS vary from changes in host-microbiome diversity leading to dysbiosis and persistent autoimmune or inflammatory stimuli, the persistence of viral reservoirs in specific tissues, alterations in the coagulation cascade, or a complex combination of multiple mechanisms (26).

In our study, any degree of hair loss was reported by 60% of PCS patients. The most accepted mechanism for alopecia is telogen effluvium (TE), which is associated with systemic stress, although other mechanisms have been described (27). It has been reported in different proportions in post-COVID patients, and some studies report a higher prevalence in women than in men, as we found, even in the sequelae initially described in patients from Wuhan, China, at the beginning of the pandemic (28, 29). Of note, arthralgias and myalgias were frequent. A physical examination and inflammatory marker determination would clarify whether arthritis occurs as a PCS manifestation or whether another condition may be unmasked. The descriptive nature of this study is a major limitation.

Our patients with PCS reported smoking more frequently, which has been described in a study from France, where smoking was the main factor associated with tachycardia or hypertension 2 months post-COVID-19 (30). Studies from the UK and Turkey found smoking was more frequently reported in patients with post-COVID-19 symptoms (31, 32). These findings underscore the importance of focusing on strategies to quit tobacco consumption among vulnerable patients as a measure to reduce PCS.

We did not find other previously described differences between groups, such as female sex, obesity, or older age. However, this study involved more than 600,000 people and included both hospitalized and outpatients, perhaps leading to a mix in various extents of viral damage, symptom duration, and baseline clinical features (31).

Regarding COVID-19 vaccination, we found no differences between our groups. However, the proportion of vaccinated patients was small at the time of the study. A systematic review by Notarte et al. (33) showed a reduction of PCS after vaccination, although this finding remains controversial with a lack of evidence to make conclusions.

A high proportion of our patients with PCS had a severe acute COVID-19 episode, although ICU stay was not associated with increased PCS. This has been inconsistently seen in studies. Kamal et al. observed that the severity of COVID-19 was related to post-COVID-19 manifestations, although 80% of patients with PCS had mild COVID-19 (5, 34, 35).

The only inflammatory marker that we found associated with PCS was a higher fibrinogen level. Fibrin amyloid micro-clotting and platelet dysfunction have been demonstrated in PCS models, unveiling a possible association between coagulation dysregulation and chronic COVID-19 symptoms (36).

A much-anticipated effect of antivirals, such as remdesivir, is the ability to protect from or ameliorate symptoms of PCS. A prospective cohort showed a 35.9% reduction of PCS at a 6-month follow-up in patients receiving remdesivir (37). Antivirals may halt the cytokine response and inflammatory cascade that activate clotting and fibrosing factors playing a role in the pathogenesis of PCS. In addition, tissue damage inflicted by SARS-CoV-2 has been linked to chronic sequelae and manifestations of organ dysfunction even months after resolution (26).

Quality of life was significantly affected in patients with PCS. This finding is consistent with Muñoz-Corona et al., who found that 75.9% of PCS patients had the lowest scores in the roles of physical dimension and general health dimension (SF-36 questionnaire) studied 90 days after discharge (17); Tobada et al. showed a decrease in QoL measured with the EQ-5D 6 months after the acute infection with moderate-to-severe disturbances in the following domains: 56% in mobility, 48% in pain/discomfort, 46% in anxiety/depression, 37% in usual activities, and 13% in self-care; these findings are similar to ours (38). In addition, a systematic review confirmed that QoL in PCS patients was significantly affected, regardless of time elapsed since discharge or recovery, although the tools applied to measure QoL were heterogeneous (39, 40). Thiolliere et al. (41) compared the QoL of older patients with COVID-19 who required ICU with other ICU patients and found no differences between the EQ-5D scores or autonomy at day 180. This finding supports that in older people, the deterioration of QoL is more likely linked to the infection per se and not to the ICU stay (41).

Our study has various limitations: The cross-sectional design does not allow for follow-up data at different times. However, the survey was conducted at different times after the acute COVID-19 episodes. Although memory bias was likely present, the survey strategy was the most efficacious approach to surpass the decline in COVID-19 cases over time, for reasons such as the circulation of variants and the current use of vaccines. In addition, none of the patients had a QoL assessment before their acute COVID-19, so direct comparisons cannot establish a strong causal relationship with SARS-CoV-2 infection. On the other hand, the patient's clinical characteristics and comorbidities were fully analyzable, which gives our results and study strength when compared with other works. Finally, a prospective approach with clinical evaluation and intervention is undoubtedly required for these patients, from the moment they are diagnosed with COVID-19, to assess the development of PCS. We consider the descriptive nature of our approach to be a limitation since we did not have any studies or interventions in those who had PCS. During the pandemic, our institution focused solely on caring for patients with COVID-19. However, due to these findings, we are currently developing multidisciplinary care for patients with PCS.

Regarding the diagnosis and management of PCS, it took not long for the scientific community to understand the complexity of PCS. Thus, since early 2021, several multidisciplinary programs and ambulatory rehabilitation clinics and projects have been launched, whether virtual or hybrid, to be able to cope with this ever-growing population. Diagnostic criteria have been set through a Delphi consensus (2). However, treatment strategies are still under investigation, mainly to ascertain the best type and duration of therapy necessary for a patient suffering from PCS to restore health and QoL (42).

Although in the future, the prevalence and severity of PCS will be modified by factors such as more robust vaccination schemes, antivirals, or anticoagulants. Furthermore, infections with new viral strains and host-derived factors may impact PCS incidence (43). Comprehensive multidisciplinary studies are needed to set the ground for better understanding and managing this disease.

## Conclusion

A high prevalence of PCS in previously hospitalized patients with COVID-19 was found. Smoking, severe COVID-19, lower SatO2 on admission, increased lung involvement, and elevated fibrinogen levels were associated with increased frequency of PCS. Some symptoms, such as difficulty concentrating, arthralgia/myalgia, and hair loss, were more prevalent in women with PCS. A significant QoL impairment was evident in PCS.

## Data availability statement

The original contributions presented in the study are included in the article/supplementary material, further inquiries can be directed to the corresponding author.

## Ethics statement

The studies involving human participants were reviewed and approved by Ethics and Research Committee, Instituto Nacional de Ciencias Médicas y Nutrición “Salvador Zubirán”. The patients/participants provided their written informed consent to participate in this study.

## Author contributions

YF-S conducted the surveys. CR-M, YF-S, and GG-B performed the clinical data search and capture. CR-M, YF-S, GG-B, KT-T, and MG-L aided in interpreting the results and worked on the manuscript. All authors discussed the results and commented on the manuscript. MG-L, JS-O, and AL supervised the research, provided critical feedback, and helped shape the research, analysis, and manuscript. All authors participated in the idea, general objectives, and design of the study.
